# The tocopherol transfer protein mediates vitamin E trafficking between cerebellar astrocytes and neurons

**DOI:** 10.1016/j.jbc.2022.101712

**Published:** 2022-02-09

**Authors:** L. Ulatowski, Mikel Ghelfi, Ryan West, J. Atkinson, C.J. Finno, D. Manor

**Affiliations:** 1 Department of Biology, Ursuline College, Pepper Pike, Ohio, USA; 2 Department of Chemistry, Brock University, St Catharines, Ontario, Canada; 3 Department of Population Health and Reproduction, University of California School of Veterinary Medicine, Davis, California, USA; 4 Departments of Nutrition and Pharmacology, School of Medicine, Case Western Reserve University, Cleveland, Ohio, USA; 5 Case Comprehensive Cancer Center, Cleveland, Ohio, USA

**Keywords:** tocopherol, vitamin E, lipid trafficking, tocopherol transfer protein, lipid transport, ALS, amyotrophic lateral sclerosis, ApoE, apolipoprotein E, AVED, ataxia with vitamin E deficiency, CNS, central nervous system, DCF, dichlorodihydrofluorescein, DMEM, Dulbecco's modified Eagle's medium, FBS, fetal bovine serum, GFAP, glial fibrillary acidic protein, HBSS, Hanks' balanced salt solution, Lrp1, low-density lipoprotein receptor–related protein 1, NBD, nitrobenzoxadiazole, TTP, alpha-tocopherol transfer protein

## Abstract

Alpha-tocopherol (vitamin E) is an essential nutrient that functions as a major lipid-soluble antioxidant in humans. The alpha-tocopherol transfer protein (TTP) binds α-tocopherol with high affinity and selectivity and regulates whole-body distribution of the vitamin. Heritable mutations in the *TTPA* gene result in familial vitamin E deficiency, elevated indices of oxidative stress, and progressive neurodegeneration that manifest primarily in spinocerebellar ataxia. Although the essential role of vitamin E in neurological health has been recognized for over 50 years, the mechanisms by which this essential nutrient is transported in the central nervous system are poorly understood. Here we found that, in the murine cerebellum, TTP is selectively expressed in glial fibrillary acidic protein–positive astrocytes, where it facilitates efflux of vitamin E to neighboring neurons. We also show that induction of oxidative stress enhances the transcription of the *TtpA* gene in cultured cerebellar astrocytes. Furthermore, secretion of vitamin E from astrocytes is mediated by an ABC-type transporter, and uptake of the vitamin into neurons involves the low-density lipoprotein receptor–related protein 1. Taken together, our data indicate that TTP-expressing astrocytes control the delivery of vitamin E from astrocytes to neurons, and that this process is homeostatically responsive to oxidative stress. These are the first observations that address the detailed molecular mechanisms of vitamin E transport in the central nervous system, and these results have important implications for understanding the molecular underpinnings of oxidative stress–related neurodegenerative diseases.

Vitamin E is a collective term that denotes a family of eight neutral plant lipids (1), of which α-tocopherol is selectively retained in the body and considered the most biologically active form of the vitamin (2, 3). The potent radical-trapping activity of α-tocopherol is thought to prevent free radical–induced lipid peroxidation, lending the vitamin its distinction as the major lipid-soluble antioxidant in most species. Consequently, adequate vitamin E status is considered an essential line of physiological defense against oxidative stress–related diseases.

The essential requirement for α-tocopherol for maintaining central nervous system (CNS) health has been recognized for over 50 years. Secondary vitamin E deficiency accompanying fat malabsorption diseases, such as abetalipoproteinemia, cholestasis, and short bowel syndrome, manifests in CNS dysfunctions that can often be remedied by timely supplementation with α-tocopherol (4, 5, 6, 7, 8, 9). The unique metabolic profile of the CNS, coupled with high levels of polyunsaturated fatty acids in neurons and astrocytes, renders this tissue particularly vulnerable to lipid peroxidation and subsequent cellular damage. Indeed, elevated levels of oxidative stress markers accompany a variety of neuropathological conditions. Analyses of autopsy samples from human patients afflicted with Alzheimer's disease (10, 11), Parkinson's disease (12), and amyotrophic lateral sclerosis (ALS) (13) reveal elevated levels of oxidative stress markers, especially lipid peroxidation products. Moreover, α-tocopherol concentrations in plasma and cerebrospinal fluid from Alzheimer's disease and ALS patients are significantly lower than in healthy individuals (14, 15, 16, 17, 18, 19). In early studies, supplementation with vitamin E was shown to decrease the levels of oxidative stress markers and to attenuate disease progression in human patients and in transgenic models of Alzheimer's disease, Parkinson's disease, and ALS (19, 20, 21, 22, 23, 24, 25, 26, 27, 28). These findings laid the foundation for prescribing α-tocopherol supplements to patients afflicted with related disorders (23, 24, 29). However, because of conflicting results from more recent studies (30, 31), the utility of vitamin E as a preventative or therapeutic measure in neurodegenerative diseases remains unclear (32).

In the liver, the alpha-tocopherol transfer protein (TTP) facilitates the secretion of dietary α-tocopherol from hepatocytes to circulating lipoproteins for delivery to nonhepatic tissues, thereby regulating whole-body status of the vitamin (33, 34, 35). The critical role of TTP is underscored by the fact that mutations in the *TTPA* gene cause the familial disorder ataxia with vitamin E deficiency (AVED; Online Mendelian Inheritance in Man: 277476; (36, 37, 38)). AVED is characterized by low plasma tocopherol levels, elevated levels of oxidative stress markers, and presents as progressive spinocerebellar ataxia, dysarthria, areflexia, and neuropathy (38, 39, 40). Despite the fact that the only described outcomes of TTP mutations are neurological, and TTP is expressed in CNS (41, 42), very little is known regarding the specific roles and mechanisms of action of TTP in the CNS. We present here our findings on the expression of TTP in cerebellar astrocytes and its role in maintaining vitamin E adequacy in neurons under normal conditions as well as those of oxidative stress.

## Results

### Expression of TTP is spatially and temporally regulated in the mouse brain

**Figure 1:**
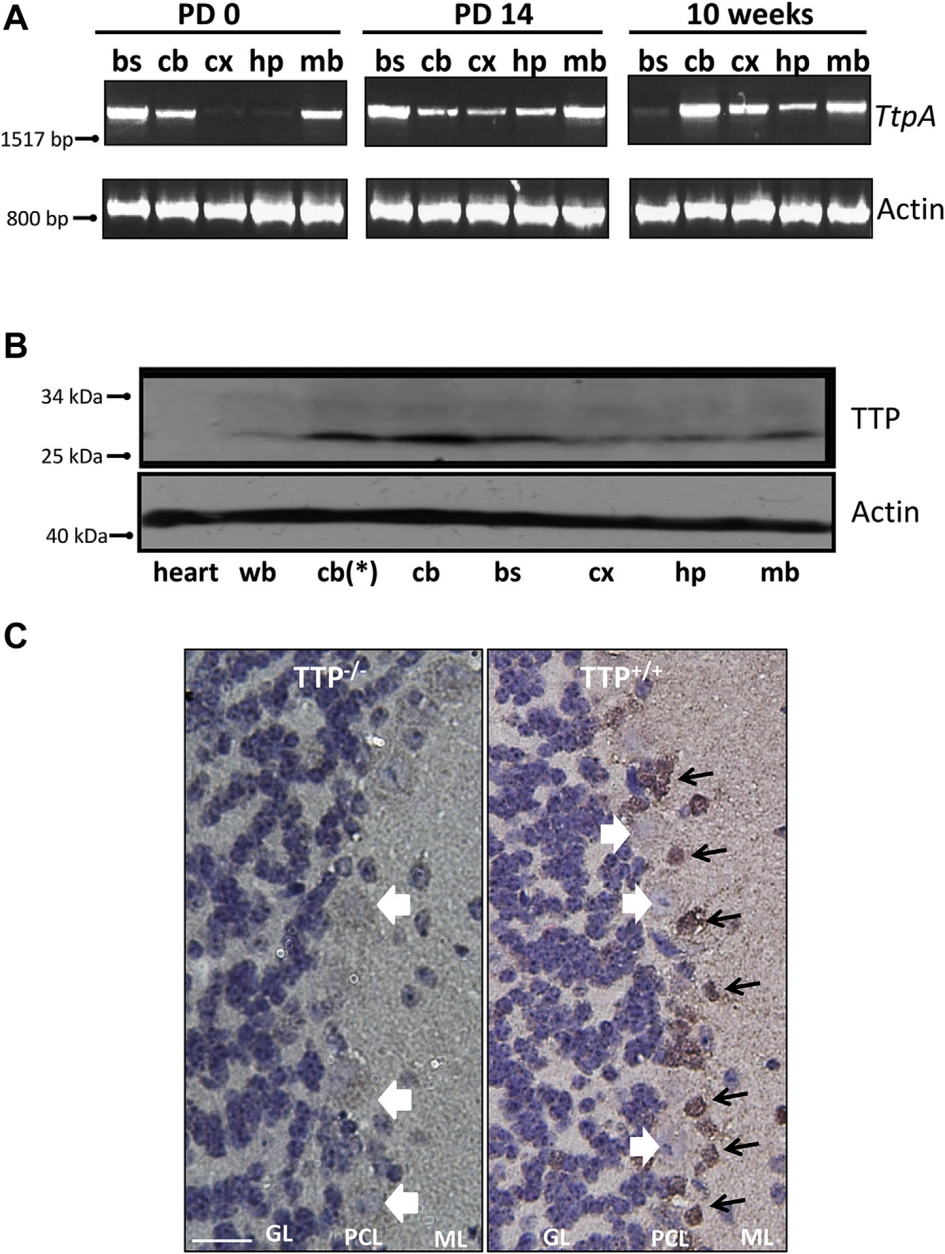
**Expression of TTP in the brain varies spatially and temporally and is restricted to astrocytes in the cerebellum.***A*, expression of TTP mRNA in the mouse brain. Tissue lysates from indicated brain regions of 10-week-old C57BL6J WT mice were used to determine mRNA levels of *TtpA* by RT–PCR. *B*, expression of TTP protein in the mouse brain. Tissue lysates from indicated brain regions of 10-week-old C57BL6J WT mice (except where indicated by *asterisk*; 8 days old) were analyzed by anti-TTP immunoblotting. Two hundred micrograms of protein of the indicated tissues were used. Heart extract is shown as a negative control. Mice were 10 weeks old (except the cerebellum samples, which were prepared from 8-day-old mice). *C*, anti-TTP immunohistochemistry staining of paraffin-embedded C57BL6J WT (*left*) and *Ttpa*^−/−^ (*right*) mouse cerebella sections counterstained with hematoxylin. The *black arrows* demarcate the positive TTP signal, whereas the *bold white arrows* point to the Purkinje neuron soma. The scale bar represents 20 μm. B, cerebellum; BS, brainstem; CX, cerebellar cortex; GL, granule cell layer; HP, hippocampus; MB, midbrain; ML, molecular layer; PCL, Purkinje cell layer; TTP, alpha-tocopherol transfer protein; WB, whole brain.

The roles of TTP in regulating tocopherol transport have been primarily studied in its site of highest expression, the liver (35, 43). However, the protein is also highly expressed in the brain, kidney, lung, and placenta (41, 42, 44, 45), where its functions are poorly understood. As AVED results in strictly neurological consequences (36, 38), we focused our studies to the CNS, specifically the brain. Using semiquantitative real-time RT–PCR, we found that the murine *TtpA* mRNA is expressed in select regions of the brain, and that this expression pattern changes dramatically with age. Thus, expression of *TtpA* in the brainstem is highest in newborn pups but is almost undetectable at 10 weeks (Fig. 1*A*). Expression in the cortex and hippocampus is very weak at birth but increases gradually with age. Expression of the *TtpA* mRNA in the cerebellum is detectable at all ages and is highest among all brain regions in the mature animal (Fig. 1*A*, (41)). Importantly, the expression profile of the TTP mRNA in the mature mouse brain (Fig. 1*A*) parallels that of the TTP protein (Fig. 1*B*). These data support and extend our previous results (46), indicating that expression of TTP is spatially and temporally regulated.

The high expression of TTP in the cerebellum (Fig. 1*A*) and our previous findings of degeneration of cerebellar Purkinje neurons in TTP-null mice (47) align with the cerebellar deficits presented by AVED patients (*e.g.*, (36, 38, 48)). To address the function of TTP in the cerebellum, we first determined the distribution pattern of the protein in the murine cerebellum using specific anti-TTP immunohistochemistry. We observed strong immunoreactivity in cerebella from WT mice but not in tissues obtained from TTP-null animals (Fig. 1*C*). Expression of the TTP protein in the cerebellum was restricted to small cells surrounding the Purkinje neurons, most likely Bergman glia (Fig. 1*C*; (41)).

### *Ttpa* is selectively expressed in cerebellar astrocytes

**Figure 2:**
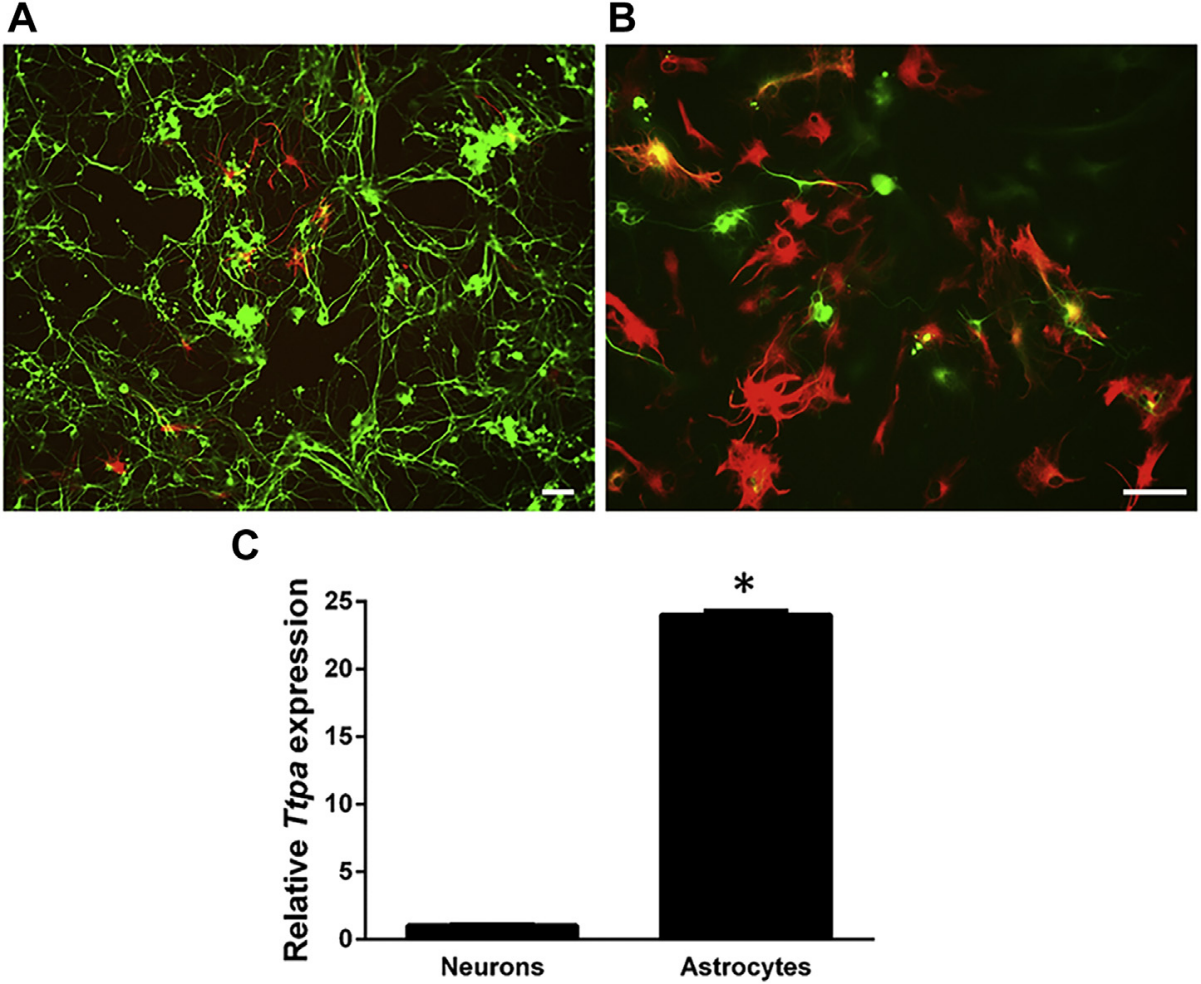
**Ttpa is preferentially expressed in cerebellar astrocytes.** Primary dissociated cerebella cells harvested from 8-day-old C57BL6J WT mice were maintained in culture as described in the Experimental procedures section for 2 days (*A*) and 8 days (*B*) prior to immunostaining with the neuronal marker β-tubulin III (*green*) and the astrocyte-specific marker GFAP (*red*). The scale bars represent 20 μm. *C*, expression of TTP mRNA was measured in astrocyte-enriched or neuron-enriched cerebellar cultures using real-time RT–PCR. Expression was normalized to 18 s. Error bars indicate standard deviations, and *asterisk* denotes *p* > 0.05. GFAP, glial fibrillary acidic protein; TTP, alpha-tocopherol transfer protein.

**Figure 3:**
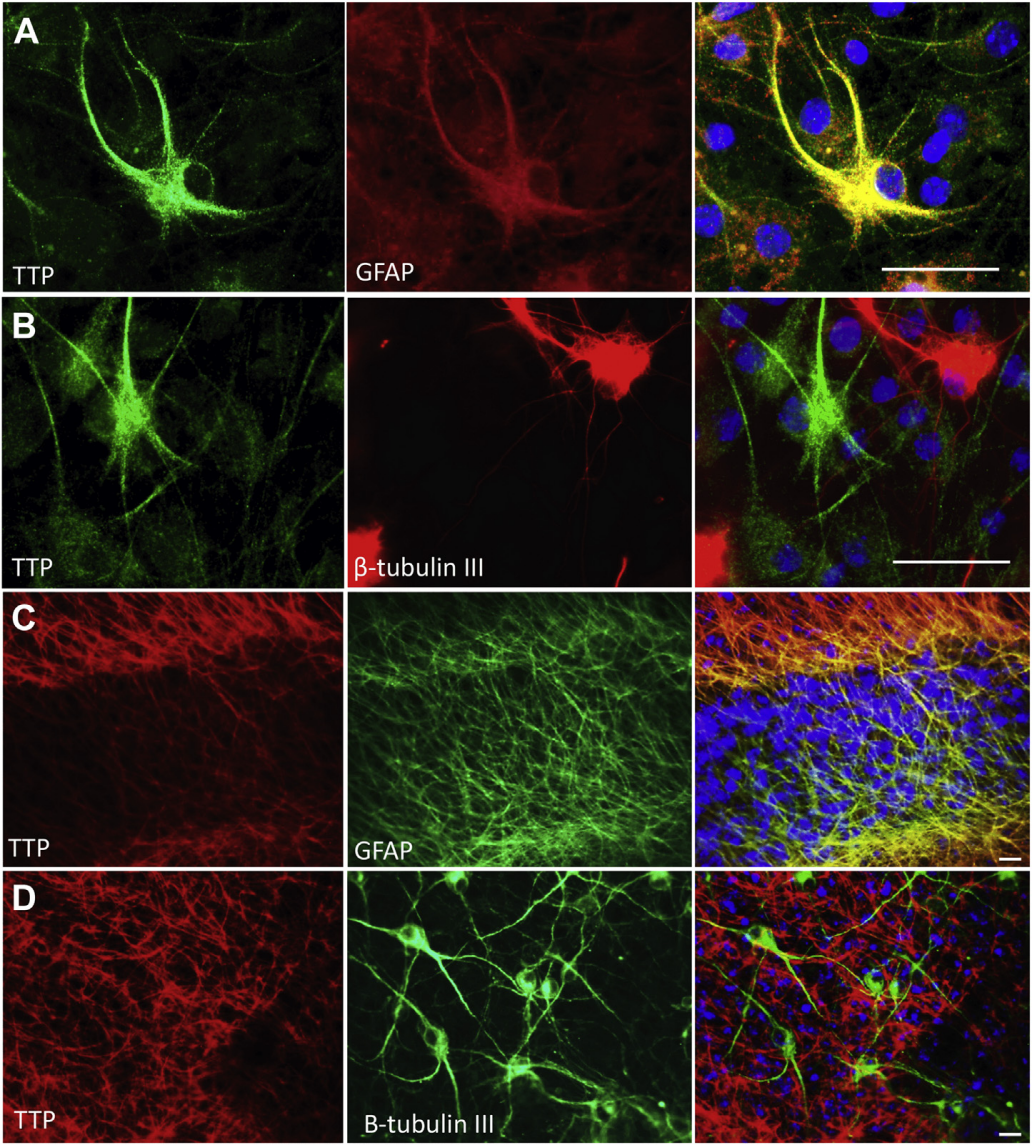
**TTP is expressed in astrocytes.** Primary dissociated astrocyte-enriched (*A*) and neuron-enriched (*B*) cerebellar cultures and cerebellar organotypic slice cultures (*C* and *D*) harvested from an 8-day-old C57BL6J WT mice were maintained in culture prior to immunofluorescence with antibodies against TTP (*A* and *B*; *green* or *C* and *D*; *red*) and the astrocyte marker GFAP (*A* and *C*; *red*) or the neuronal marker β-tubulin III (*B* and *D*; *red*). Note the *yellow* in the merged images of (*A* and *C*) indicates colocalization. DAPI (*blue*) staining identifies the nuclei. The scale bars represent 20 μm. DAPI, 4′,6-diamidino-2-phenylindole; GFAP, glial fibrillary acidic protein; TTP, alpha-tocopherol transfer protein.

The CNS is composed of three main cell populations; neurons, microglia, and macroglia. Macroglia, in turn, can be further distinguished into astrocytes and oligodendrocytes, based on their precursor cells. To identify the cell type(s) in which TTP is expressed, we cultured dissociated cerebella and employed culture conditions that selectively enrich astrocyte or neuronal cell populations (see Experimental procedures section). We then stained each culture with antibodies against an established astrocyte-specific marker (glial fibrillary acidic protein [GFAP]; (49, 50)) or a neuronal-specific marker (β-tubulin III; (51, 52, 53)). Immunofluorescence staining confirmed that the respective culture conditions indeed yielded cell populations that were highly enriched (>90%) in neurons or astrocytes (Fig. 2, *A* and *B*, respectively). We then determined which of these cell types express TTP. Real-time RT–PCR revealed that *Ttpa* is expressed at much higher levels (>24-fold) in astrocytes as compared with neuron-enriched cultures (Fig. 2*C*). These findings are supported by a published expression profile in which *Ttpa* expression was shown to be high in murine astrocytes but not in neurons or oligodendrocyte cell populations (54). We next examined the localization of the TTP protein in cultured dissociated cerebella, where all cell types are present. Using immunofluorescence, we found that TTP is expressed in GFAP-positive astrocytes, where it is distributed throughout the cytoplasm and dendrites (Fig. 3*A*). We did not detect any TTP expression in β-tubulin III–positive neurons (Fig. 3*B*). Finally, we examined the expression pattern of TTP in organotypic slice preparations, where the cellular microenvironment, tissue architecture, and electrochemical characteristics of individual cells are preserved (55, 56). Again, we observed strong staining of TTP in GFAP-positive cells but not in neurons (Fig. 3, *C* and *D*). Quantitative analyses of the merged images showed a strong overlap between the localization of TTP and GFAP (Pearson's correlation coefficient, *r* = 0.55), whereas localization of TTP in β-tubulin III–expressing cells was not significant (Pearson's correlation coefficient, *r* = 0.03). Taken together, our data provide strong evidence that TTP mRNA and protein is selectively expressed in GFAP-positive astrocytes and not in neurons.

### Vitamin E is selectively stored in TTP-expressing astrocytes

**Figure 4:**
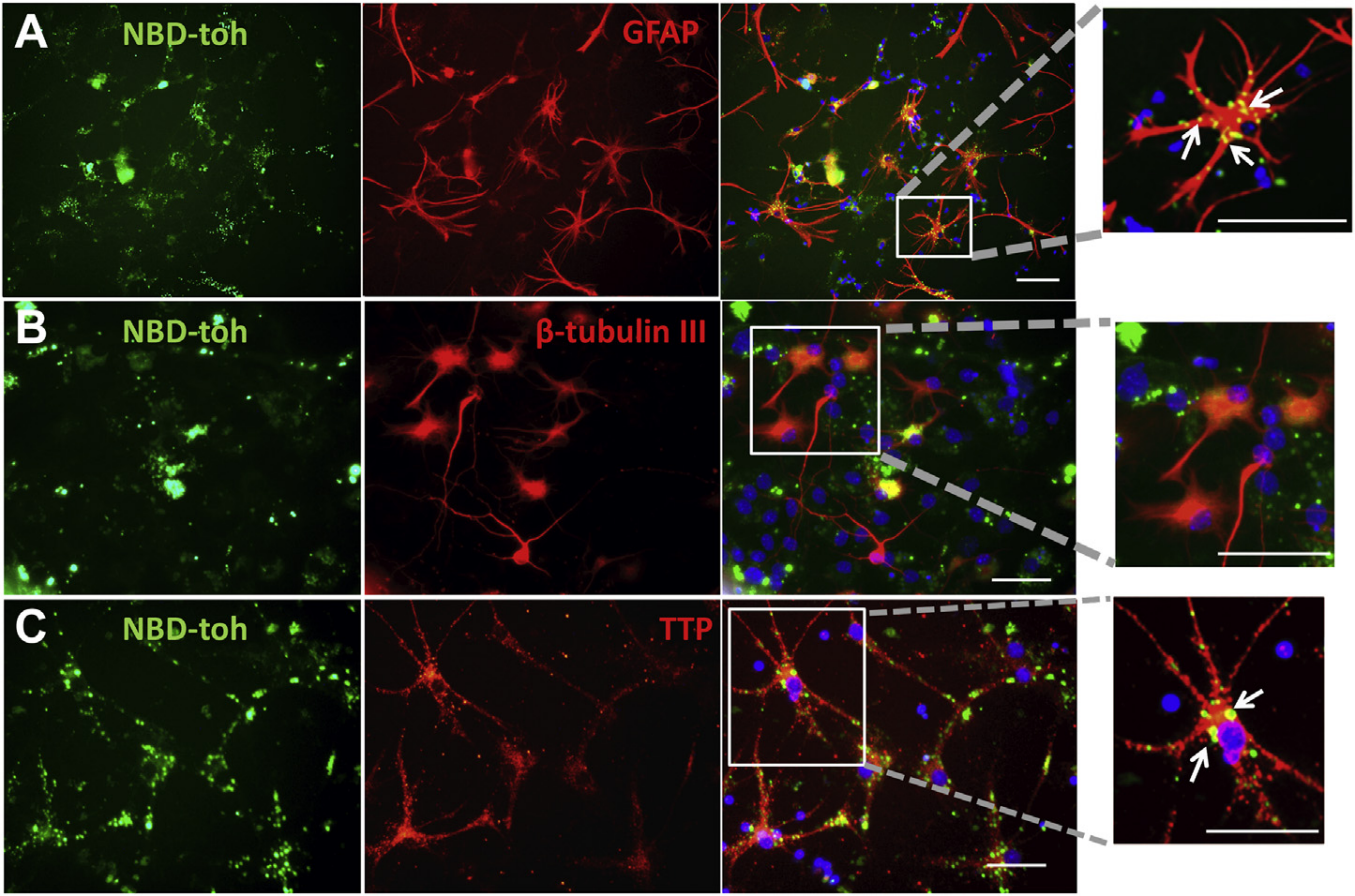
**Vitamin E preferentially localizes to GFAP-positive and TTP-expressing cerebellar astrocytes.** Merged images of primary dissociated cerebella cultures harvested from 8-day-old WT mice maintained in culture for 6 days prior to “loading” with NBD-tocopherol (*green*) and coimmunostaining with the astrocyte marker GFAP (*red*; *A*), the neuronal marker β-tubulin III (*red*; *B*), or TTP (*red*; *C*). *White arrows* in inset images indicate the NBD-tocopherol colocalization. The scale bars represent 20 μm. GFAP, glial fibrillary acidic protein; NBD, nitrobenzoxadiazole, TTP, alpha-tocopherol transfer protein.

We next determined the cell type where α-tocopherol accumulates in the mouse cerebellum. To determine the uptake and distribution of the vitamin, we employed nitrobenzoxadiazole (NBD)-tocopherol, a fluorescent analog of vitamin E that we have previously shown to be a faithful reporter of vitamin E *in vitro* and *in vivo* (34, 57, 58, 59, 60, 61). Since tocopherol is thought to enter cells bound to lipoprotein particles *via* specific surface receptors (34, 43, 62, 63, 64, 65, 66, 67, 68), we delivered NBD-tocopherol complexed to serum lipoproteins (33, 69, 70) or to apolipoprotein E (ApoE; (71, 72)). After overnight “loading” with the fluorescent vitamin, cultured dissociated cerebella were stained and visualized by confocal fluorescence microscopy. Strong NBD-tocopherol fluorescence was observed in GFAP-positive astrocytes (Fig. 4*A*), as compared with β-tubulin III–positive neurons, where NBD fluorescence was much weaker (Fig. 4*B*). Importantly, NBD-tocopherol fluorescence was especially evident in TTP-positive cells (Fig. 4*C*). These observations suggest that TTP-expressing astrocytes accumulate vitamin E, and that expression of TTP in these cells regulates the vitamin's distribution in the brain.

### Vitamin E is localized to lysosomes and mitochondria

**Figure 5:**
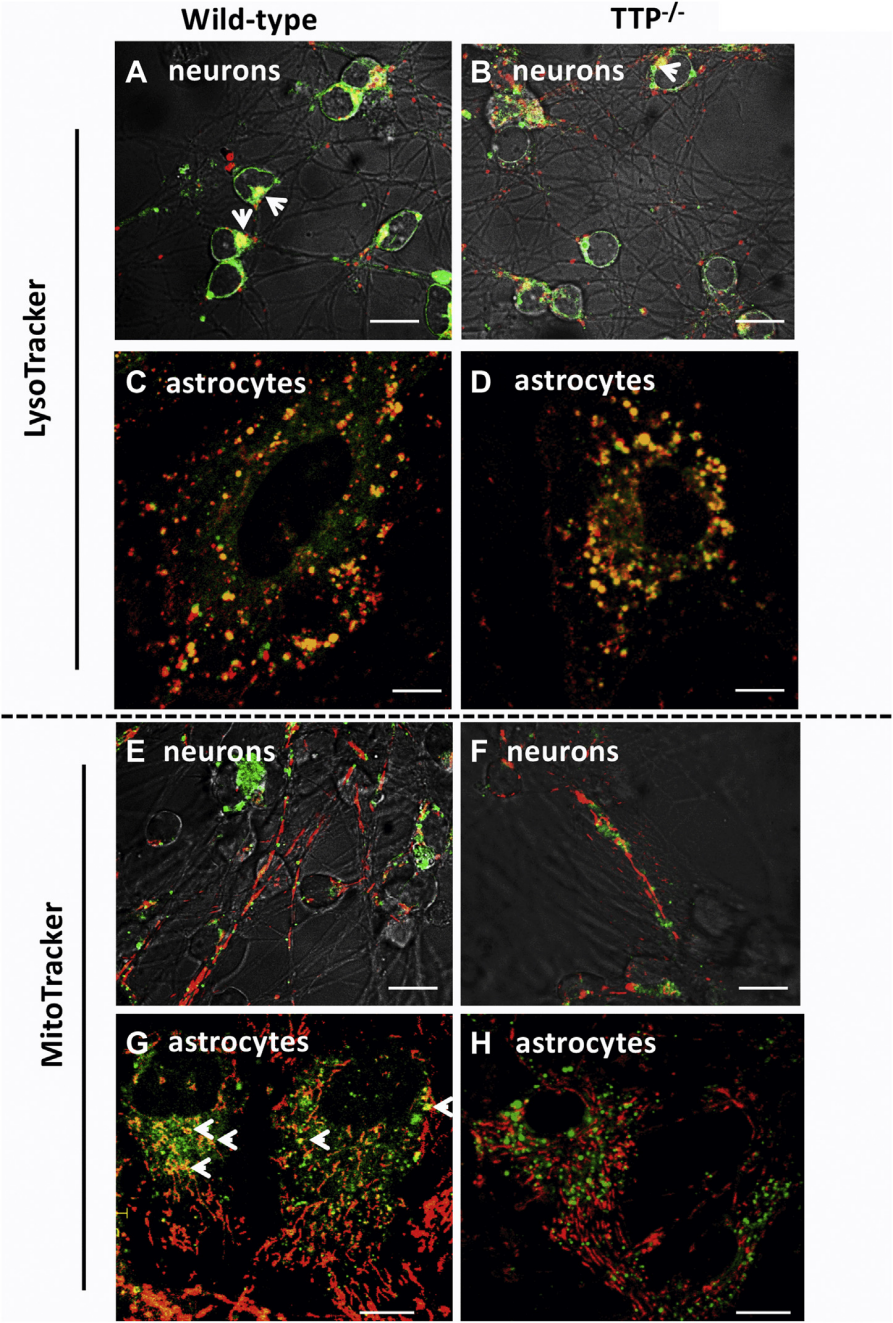
**Vitamin E localizes to lysosomes and mitochondria in primary neurons and astrocytes and is not affected by the presence of TTP.** Merged images of *Ttpa*^*+/+*^ primary dissociated neuron (*A* and *B*) and astrocyte (*C* and *D*) cerebella cells maintained in culture for 24 h and 7 days, respectively, from *Ttpa*^*+/+*^ (*A*, *C*, *E*, and *G*) and *Ttpa*^−/−^ (*B*, *D*, *F*, and *H*) mice prior to an overnight loading with BODIPY-tocopherol (*green*) and a subsequent staining with LysoTracker Red (*A* and *C*; *red*) to mark the lysosomes or with MitoTracker Red (*B* and *D*; *red*) to visualize mitochondria. The *yellow* in the perinuclear region of the neurons (*A*) and astrocytes (*C*) indicates localization of BODIPY-tocopherol to lysosomes. The *yellow* in the overlaid images indicates that BODIPY-tocopherol localizes to mitochondria in the astrocytes (*D*) but not in the neurons. These findings were unchanged in *Ttpa*^−/−^ mice (*B*, *D*, *F*, and *H*), indicating that this distribution pattern is not affected by the presence of TTP. The scale bars represent 10 μm. TTP, alpha-tocopherol transfer protein.

The intracellular localization of vitamin E may provide important insights into the mechanisms that regulate its actions. Analytical determinations of vitamin E distribution in liver revealed highest concentrations in lysosomes (73, 74) and mitochondria (75), but the location of vitamin E within cells of the brain has not been reported. To determine the compartment where vitamin E accumulates, we loaded neuron-enriched or astrocyte-enriched primary cerebellar cultures with BODIPY-tocopherol and visualized the intracellular localization of the vitamin as compared with established organelle markers. Our observations indicate that (1) under all conditions tested, the fluorescent tocopherol accumulated at higher levels in astrocytes as compared with neurons (Fig. 5); (2) in both neurons and astrocytes from WT mice, we observed extensive colocalization between the fluorescent vitamin and the lysosomal marker LysoTracker (Fig. 5, *A* and *C*), suggesting that entry of vitamin E into astrocytes and neurons occurs through the endocytic pathway; (3) BODIPY-tocopherol fluorescence also exhibited moderate overlap with the mitochondria-selective marker MitoTracker in astrocytes (Fig. 5*G*) and minor mitochondrial colocalization in neurons (Fig. 5*E*); (4) the intracellular distribution pattern of BODIPY-tocopherol was not altered in cerebellar cells prepared from TTP-null mice. Thus, in TTP^−/−^ cells, BODIPY-tocopherol accumulated preferentially in astrocytes, where it colocalized with lysosomes (Fig. 5, *B* and *D*) but to a much lesser extent with mitochondria (Fig. 5, *F* and *H*). These observations show that the presence of TTP does not affect uptake of lipoprotein-complexed vitamin E. Rather, TTP may regulate vitamin E trafficking by modulating secretion of α-tocopherol from the cells, as it does in hepatocytes (34).

**Figure 6:**
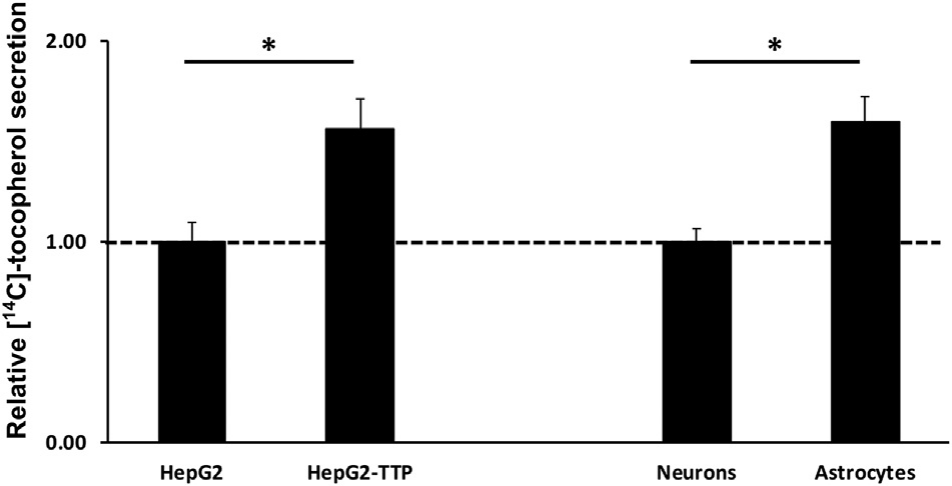
**Secretion of vitamin E is enhanced in TTP-expressing hepatocytes and astrocytes.** HepG2-TetOn-TTP cells (*left panel*) were induced to express TTP with doxycycline (1 μg/ml) where indicated. Primary cerebellar neurons and astrocytes (*right panel*) were harvested from *TtpA*^+/+^ mice and cultured as described in the Experimental procedures section. Cells were loaded for 48 h with [^14^C]-α-tocopherol, followed by washes, and a secretion period of 24 h. *Asterisks* denote significant difference in secretion between the neurons and astrocytes or the induced and uninduced HepG2 cells (*p* < 0.05; Student's *t* test). TTP, alpha-tocopherol transfer protein.

To directly test this possibility, we loaded cells with [^14^C]-α-tocopherol and measured secretion of the labeled vitamin into the culture media over time using a previously established assay (34). We found that astrocytes secreted vitamin E to a greater extent (approximately twofold) than neurons under identical conditions (Fig. 6). Notably, the magnitude of TTP-induced vitamin E secretion from astrocytes was similar to that observed in TTP-expressing hepatocytes, where the physiological function of TTP is established (Fig. 6; (34)). To complement these findings, we examined the effect of astrocyte-TTP on the intracellular levels of the vitamin. We loaded astrocytes with NBD-tocopherol and monitored its fluorescence within cells over time. Four hours after loading, intracellular fluorescence from NBD-tocopherol decreased by 30% (Table 1). As we have previously shown, this change in fluorescence reflects secretion of the fluorescent vitamin from the astrocytes to the culture media (34, 58, 60, 61, 76, 77). Importantly, no vitamin E was secreted from astrocytes prepared from TTP^−/−^ mice (Table 1), indicating that this process is TTP dependent. Taken together, these findings show that trafficking of vitamin E within the cerebellum involves preferential accumulation of vitamin E in astrocytes, and TTP-dependent secretion from these cells, likely to neighboring neurons.

**Table 1:** Intracellular accumulation of NBD-tocopherol in cultured cerebellar astrocytes

Astrocytes source	ΔF/F (%)^a^
WT	−30%
TTP^−/−^	ND
WT + glyburide	+220%
TTP^−/−^ + glyburide	+270%

Cultured cerebellar astrocytes were loaded with ApoE-complexed NBD-tocopherol as described in the Experimental procedures section.

ND, below our detection limit of 3% fluorescence change; *p* < 0.05.

Abbreviation: ND, not detectable.

a NBD fluorescencewas quantified by fluorescence imaging, and changes in intracellular NBD fluorescence during 4 h of secretion were quantified from 10 microscopic fields. Average values ± standard deviations are shown. To investigate the role of ABC transporters in this process, glyburide, an established inhibitor of ABC-type transporters, was added to 10 μM before loading.

### Transport of α-tocopherol between astrocytes and neurons

Next, we aimed to determine which other proteins participate in the transport of vitamin E between astrocytes and neurons. Since several transmembrane transporters (*e.g.*, ABCA1, ABCG1, ABCG4) are expressed in the brain and mediate trafficking of cholesterol between different cell types (78, 79, 80, 81, 82), we examined their role in vitamin E transport in cultured cells. Thus, we monitored the secretion of vitamin E in cultured astrocytes in the presence and absence of glyburide, an established inhibitor of ABC-type transporters (82). We loaded cerebellar astrocytes with NBD-tocopherol and monitored the time-dependent change in intracellular fluorescence as described previously. We observed a twofold to threefold increase in intracellular NBD fluorescence in glyburide-treated cells compared with control cultures (Table 1). These findings indicate that TTP-facilitated efflux of vitamin E from astrocytes is mediated by ABCA1, ABCG1, ABCG4 or a combination thereof. We also aimed to determine the transporter that mediates vitamin E uptake by neurons. We found that partial knockdown of the low-density lipoprotein receptor–related protein 1 (Lrp1) (79, 81) in differentiated SY5Y neurons inhibited NBD-tocopherol uptake by 15 to 20% (Fig. S1). Taken together, our findings suggest that vitamin E effluxes out of astrocytes in a pathway that requires TTP and an ABC-type transporter, and that uptake by neurons involves the Lrp1 receptor. This model is very similar to that established for the transport of cholesterol between astrocytes and neurons (83, 84).

### Regulation of TTP expression by oxidative stress

**Figure 7:**
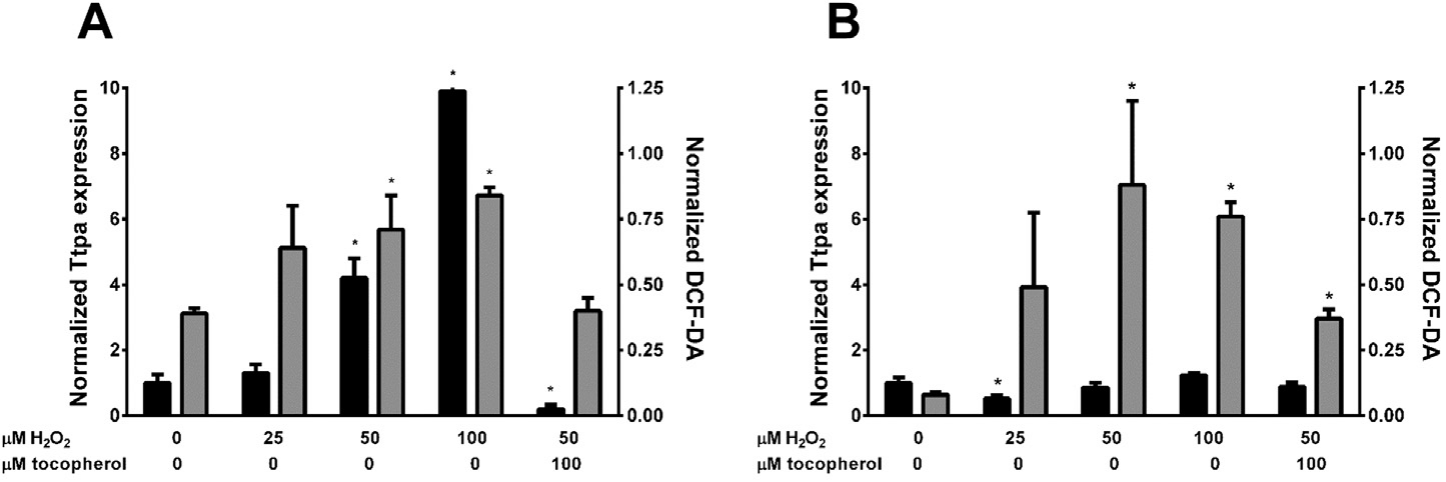
**Expression of the *TtpA* gene in cerebellar astrocytes is regulated by oxidative stress.** Real-time PCR results of normalized *Ttpa* expression (*black bars*; *left axis*) and H_2_O_2_-induced oxidative stress, as determined by the DCF-DA assay (*gray bars*; *right axis*), in primary astrocytes (*A*) or neurons (*B*). Where indicated, tocopherol was added 16 h prior to H_2_O_2_ addition. *Asterisks* indicate significance of *p* < 0.05 compared with nontreated cells (one-way ANOVA with Tukey's post hoc testing). DCF-DA, dichlorodihydrofluorescein diacetate.

It is currently not known whether vitamin E trafficking in the nervous system is a continuous process or is subject to dynamic regulation by physiological parameters. We have previously shown that the expression of the *TtpA* gene in hepatocytes is modulated by oxidative stress (46). To examine whether this response occurs also in cerebellar cells, we induced oxidative stress in neuron-enriched and astrocyte-enriched cerebellar cultures with hydrogen peroxide and measured the expression of the *TtpA* mRNA using real-time RT–PCR (Fig. 7). We found that addition of H_2_O_2_ to the culture media induced a dose-dependent increase in intracellular free radical species, as measured by the cell-permeable probe dichlorodihydrofluorescein (DCF) diacetate, which reports on intracellular reactive oxygen species (85). Pretreatment of the cells with α-tocopherol eliminated the H_2_O_2_-induced DCF fluorescence, attesting to the vitamin's antioxidant properties and the validity of this cell system. In cultured cerebellar astrocytes, increased oxidative stress was accompanied by a dramatic (reaching 10-fold) dose-dependent increase in TtpA mRNA levels, which was prevented by pretreatment with vitamin E (Fig. 7*A*). In cultured cerebellar neurons, on the other hand, expression of the *TtpA* gene did respond neither to H_2_O_2_-induced oxidative stress nor to the addition of α-tocopherol (Fig. 7*B*). Our findings confirm and extend previous reports that documented enhanced expression of TTP in response to oxidative stress in hepatocytes (46), trophoblasts (86), zebrafish (87), and human brain (42).

## Discussion

The pivotal link between α-tocopherol and neurologic health in humans was made by the discovery that mutations in the *TTPA* gene cause the debilitating familial neurological syndrome AVED (37, 88). The TTP (encoded by the *TTPA* gene) has been shown to regulate body-wide levels of α-tocopherol by facilitating the secretion of the dietary vitamin from hepatocytes to circulating lipoproteins (38). A long-standing enigma has been reconciling the strict neurological phenotype of AVED with the fact that the primary site of expression of TTP is the liver, whereas only minute amounts of the protein were reported in the CNS. We therefore sought to define the role of TTP in vitamin E transport in specific cells within the brain. We focused our attention on the cerebellum since TTP is expressed in this region, it is the primary site of neurological deficits manifested in AVED patients, it is the brain region where the greatest dysregulation of gene expression is found in *Ttpa*^−/−^ mice (89) and is the most active in vitamin E metabolism (71). We found that, in the cerebellum, TTP is selectively expressed in the GFAP-positive astrocytes, where it facilitates efflux of vitamin E to neighboring neurons *via* an ABC-type transporter. Uptake of vitamin E into neurons is mediated, at least in part, by the LRP1. Finally, we found that induction of oxidative stress enhances the transcription of the *Ttpa* gene in astrocytes, thereby providing a means to maintain oxidative homeostasis in the brain in the face of increased oxidative challenge.

While TTP is most highly expressed in liver, AVED patients present with strict neurologic symptoms, and postmortem findings indicate axonal degeneration of the posterior column and mild Purkinje cell loss (90, 91). At postnatal day 0, *Ttpa* expression was highest within susceptible neuroanatomic regions of the posterior column, including the brainstem, cerebellum, and midbrain. It is important to note that molecular dysregulation and abnormal histologic changes first appear in the spinal cord of vitamin E-deficient *Ttpa*^−/−^ mice (92). Axonal degeneration continues within the spinocerebellar tract as these mice age, culminating in Purkinje cell loss by 17 to 20 months of age (47, 93). Our data show that cerebellar expression of the *TtpA* gene increases with age, paralleling the anatomic injury and the delayed-onset nature of the spinocerebellar ataxic phenotype.

Our findings confirm and extend an earlier study that suggested selective expression of TTP in cerebellar Bergmann glial cells (41). Among the subtypes of glia, astrocytes serve the most diverse roles, providing structural and metabolic support to neurons, for example, glycogen storage and uptake of ions and neurotransmitters from the synaptic cleft (94). Facilitation of vitamin E efflux from astrocytes to neighboring neurons is remarkably similar to cholesterol transport in the brain (95). In fact, secretion of α-tocopherol from astrocytes and uptake of the vitamin into neurons is mediated by the very same proteins that transport cholesterol, that is, an ABC-type transporter and the LRP1, respectively (96). Experimental perturbations to cholesterol transport in the brain were shown to cause impaired development and function of the brain (97, 98). Our data raise the possibility that vitamin E status is also disrupted in these situations and may play an important and underappreciated role in related pathologies.

**Figure 8:**
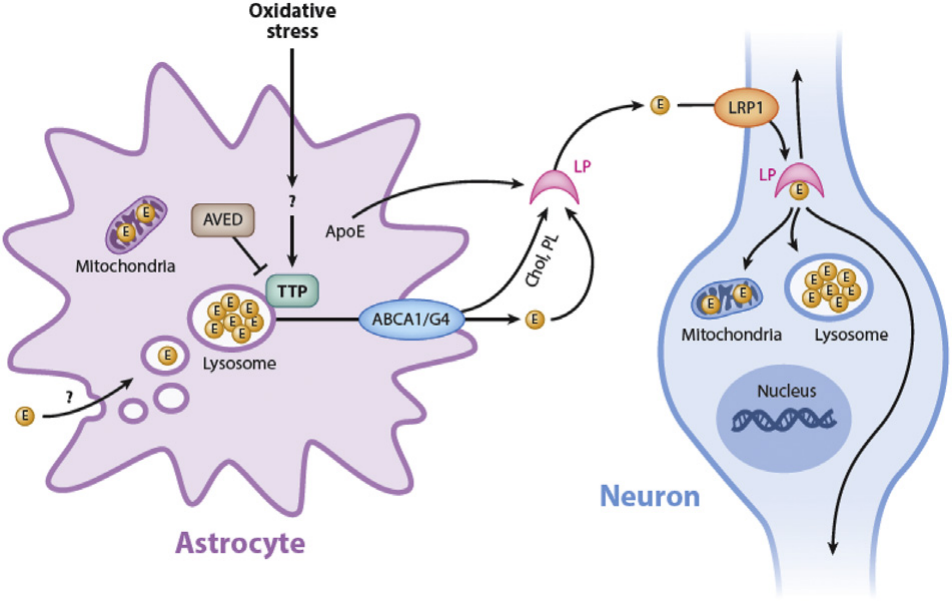
**Working model of vitamin E transport in the cerebellum.** Alpha-tocopherol accumulates in astrocytes, such as the cerebellar Bergman glia cells, where it is stored in endocytic vesicles. Astrocyte-expressed TTP facilitates the secretion of vitamin E *via* an ABC transporter to the extracellular milieu, where the vitamin is assembled into ApoE-containing lipoprotein particles, which are taken up by neighboring neurons (*e.g.*, cerebellar Purkinje cells) *via* LRP1. Elevation of oxidative stress enhances expression of TTP in astrocytes, thereby facilitating egress of the vitamin to neurons, which are especially vulnerable to oxidative stress. Loss-of-function mutations in TTP, such as those present in heritable AVED cases, impair vitamin E trafficking, thereby eliciting oxidative injury to neurons and giving rise to cerebellar ataxia. ApoE, apolipoprotein E; AVED, ataxia with vitamin E deficiency; LRP1, low-density lipoprotein receptor–related protein 1; TTP, alpha-tocopherol transfer protein.

Our findings demonstrate that levels of the *Ttpa* mRNA in astrocytes increase upon rise in intracellular oxidative stress, similar to previous observations in hepatocytes (34). Notably, transcription of *TtpA* in cerebellar neurons was not affected by oxidative stress. Since astrocytes accumulate and store vitamin E, increased TTP expression in these cells is expected to cause facilitated efflux of vitamin E to adjacent neurons. Mutated versions of TTP, as found in human AVED patients, are expected to attenuate or abolish delivery of α-tocopherol to Purkinje neurons, resulting in oxidative damage and compromised function that present as cerebellar ataxia. Results from this work and others lead to the working model of vitamin E transport in the cerebellum that is depicted in Figure 8.

Cholesterol transport in the brain is mediated by lipoprotein particles that are scaffolded around ApoE by astrocytes and used to deliver lipophilic cargo to neurons. Vatassery *et al.* (72, 99, 100) have shown that vitamin E transport in the brain utilizes ApoE particles. Importantly, the polymorphic ApoE4 variant, associated with increased risk for cardiovascular disease (101) and Alzheimer's disease (102), is associated with vitamin E status (103). It has been suggested that optimizing vitamin E content of CNS lipoproteins may be beneficial in preventing or treating Alzheimer's disease (104). Our findings support this association, as uptake of vitamin E into neurons involves the major apoE receptor in the brain, LRP1 (105). Interestingly, LRP1 levels are significantly reduced in brains of patients with Alzheimer's disease (106). As the effect of APOE4 on beta-amyloid accumulation in Alzheimer's disease is dependent on LRP1 (107), the implications of vitamin E's interaction with specific apoE alleles and the etiology of neurodegenerative diseases merit further study.

In conclusion, our data indicate that TTP-expressing astrocytes control the delivery of vitamin E from astrocytes to neurons, and that this process is homeostatically responsive to oxidative stress. These are the first observations that address the molecular mechanisms of vitamin E transport in the CNS, which may have important implications to the molecular underpinnings of oxidative stress–related neurodegenerative diseases.

## Experimental procedures

### Mice

All animal works were performed according to the Institutional Animal Care and Use Committee–approved protocols at Case Western Reserve University. The *Ttpa*^−/−^ mouse model (B6.129S4-Ttpa^tm1Far^/J; Jackson Labs) was generated by targeted disruption of exons 1 and 2 of the *Ttpa* gene and was described earlier (108). For breeding, *Ttpa*^*+/−*^ female mice were crossed with *Ttpa*^*−/−*^ males and offspring genotype determined by PCR. Animals were sacrificed at postnatal day 8, cerebella excised, and either fixed in 3.7% paraformaldehyde for 24 h followed by paraffin embedding, flash frozen, or immediately processed for primary cell cultures.

### RNA harvest and PCR

RNA was harvested using Trizol reagent (Invitrogen) followed by reverse transcription using a High Capacity cDNA Reverse Transcription Kit (Applied Biosystems). Primers for PCR amplification of *Ttpa* were 5′-CTCACAGACGCTTTCCTGCT (forward) and 5′-ACAGCACATCCCAGCTACTG’ (reverse). *Ttpa* expression was normalized to β-actin expression using the following primers: 5′-TGTGATGGTGGGAATGGGTCAGAA (forward) and 5′-TCTCCTTCTGCATCCTGTCAGCAA (reverse). For quantitative real-time RT–PCR, Taqman Fam-labeled probes for *Ttpa* (Mm00803829_m1) and 18 s (Hs99999901_s1) were used. Expression of *Ttpa* mRNA in primary cerebellar cultures was determined using comparative real-time PCR applying the Livak method of quantification (109).

### Fluorescent tocopherol analogs

The syntheses and utility of these reagents were published earlier (34, 57, 58, 59, 60, 61, 110, 111). BODIPY-tocopherol (λ_Ex_= 507 nm; λ_Em_ = 511 nm; ε = 83,000 M^−1^ cm^−1^ in ethanol; (110)) was dried under nitrogen in a siliconized tube, resuspended in 6.8 μl apoE (Athens Research & Technology; final concentration of 5 μg/ml) and 1 ml of serum-free Dulbecco's modified Eagle's medium (DMEM), and rotated overnight at 4 °C in the dark. The complexed BODIPY-tocopherol was added to the cell culture media to 20 μM. Serum-complexed NBD-tocopherol was prepared and used as described (34).

### Immunoblotting

Tissues (flash-frozen in liquid nitrogen) were lysed in radioimmunoprecipitation assay buffer (25 mM Tris [pH 7.4], 150 mM NaCl, and 1% NP-40). Endogenous expression level of TTP in lysates was determined using a rabbit polyclonal antibody raised against recombinant full-length human TTP (Covance; (112)).

### Primary cultures of cerebellar cells

Cerebella were dissected from 8-day-old C57BL/6 mice (113), minced in Hanks' balanced salt solution (HBSS) without magnesium, calcium, and phenol red (Invitrogen) and dissociated by incubation with 0.25% trypsin in PBS at 37 °C for 10 min. Triturated cells were passed through a 70 μm nylon mesh (BD Biosciences), underlayered with 2 ml of fetal bovine serum (FBS) and centrifuged at 800 RPM for 5 min. Pelleted cells were resuspended in 10% FBS/DMEM (Invitrogen) and plated on poly-l-lysine–coated plates (Sigma; 50 μg/ml) or glass coverslips.

### Enriched neuron cultures

About 24 h after plating dissociated cerebella, cell media were changed to a 1:1 mix of 10% FBS/DMEM and neurobasal media containing B27 supplement, 2 mM l-glutamine, 5 mM KCl, and 0.6% glucose. Within 48 h after harvesting, neuronal outgrowths dominated the culture, and greater than 80% of the cells stained positively for β-tubulin III.

### Enriched astrocyte cultures

About 24 h after plating dissociated cerebella, cell media were changed to 10% FBS/DMEM. The media were changed every third day, and astrocyte cell enrichment (>90%) was confirmed by staining with anti-GFAP antibodies.

### Organotypic cerebellar slice culture

The organotypic cerebellar slice culture was prepared from 8-day-old mice by a modification of established protocol for hippocampal slice cultures (114). Cerebella were removed and sagittaly sliced to 150 μm sections on a vibratome (McIlwain tissue chopper). Slices were incubated in HBSS/0.5% glucose for 30 min at 4 °C and transferred onto 0.4 μm membrane inserts (Millipore) in 6-well dishes containing 25% HBSS, 50% minimal essential medium, 25% horse serum, and 0.5% glucose. Media were changed every third day, and slices were cultured for at least 7 days.

### Knockdown of LRP1

Lentiviral shRNA constructs targeted against human LRP1 (TRCN0000053253) and a control shRNA in the pLKO vector (Thermo Scientific) were transfected into human embryonic kidney 293T cells using Lipofectamine-Plus (Invitrogen). At 24 and 48 h post-transfection, culture media from transfected dishes were pooled and pelleted by centrifugation at 100,000*g* for 1.5 h. The lentiviral pellet was resuspended in sterile PBS and used to infect cultured SH-SY5Y cells with polybrene (4 μg/ml). Infection was repeated at 48 and 72 h after the initial addition. Knockdown efficiency was evaluated using anti-LRP1 immunoblotting (catalog no.: EPR3724; Abcam).

### Immunofluorescence microscopy

Serum-complexed NBD-tocopherol (34) was loaded into primary cultures grown on 50 μg/ml poly-l-lysine–coated cover slips overnight. After washing, the cells were fixed in 3.7% paraformaldehyde, permeabilized in 5% goat serum/0.3% Triton X-100, followed by incubation with anti-TTP, anti-β-tubulin III (Sigma), or anti-GFAP (Becton Dickenson) antibodies. Goat anti-rabbit Alexa 568 (Invitrogen) was used to visualize TTP, whereas goat antimouse Alexa 488 (Invitrogen) was the secondary antibody for β-tubulin III and GFAP. 4′,6-Diamidino-2-phenylindole staining was used to visualize nuclei. After mounting in SlowFade Gold antifade reagent (Invitrogen), slides were imaged on an inverted (Leica DM4100B) or confocal (Zeiss LSM 510) microscope. Cerebellar slice cultures were immunostained according to the previous published technique (114) using the same antibodies.

### Secretion of [^14^C]-tocopherol from cultured cells

Secretion of [^14^C]-tocopherol from cultured cells was measured as previously described (34). Briefly, in 24-well poly-l-lysine–coated plates, doxycycline-treated HepG2-TetOn-TTP cells (serving as controls) or primary dissociated neuron and astrocyte cultures from C57BL/6 (WT) mice were loaded with FBS-complexed [^14^C]-*RRR*-α-tocopherol for 48 h, extensively washed, and further incubated in DMEM for the secretion period of 24 h. The secretion media were saved and pooled with the media from two subsequent washes. Finally, cells were lysed to determine the amount of cellular [^14^C]-α-tocopherol. An LS 6500 multipurpose scintillation counter (Beckman Coulter) was used to determine the amount of ^14^C-α-tocopherol in the various samples and calculate the secreted fraction of [^14^C]-α-tocopherol.

### Secretion of fluorescent vitamin E from primary astrocytes

Primary astrocytes grown in 24-well poly-l-lysine–coated plates for 7 days were loaded with apoE-complexed BODIPY-tocopherol for 16 h, washed with 10% FBS/DMEM to remove the free tocopherol, and imaged by fluorescence microscopy. Secretion was evaluated again after additional 4 h of incubation in 10% FBS/DMEM. To examine the involvement of ABC transporters, 10 μM glyburide (Sigma) was added to the cells 8 h prior to tocopherol loading. Images were taken from 10 fields in triplicate wells on a Leica DM4100B inverted microscope. Quantification of fluorescence was performed as described using ImageJ software (National Institutes of Health). Fluorescence was normalized to protein content in parallel wells using the Bio-Rad protein assay. Each experiment was repeated at least three times.

### Subcellular localization of vitamin E

Cultured primary cerebellar cells were plated on 35 mm glass-bottom poly-d-lysine–coated dishes (MatTeK Corporation) and grown as described previously for enriching in neurons or astrocytes. About 20 μM ApoE-complexed BODIPY-tocopherol were loaded for 16 h onto neurons and astrocytes after 24 h or 7 days, respectively. The cells were washed and incubated in HBSS containing either 50 nM of LysoTracker Red (Life Technologies) or MitoTracker Red (Life Technologies) to visualize lysosomes or mitochondria, respectively. Fluorescence microscopy was performed on a Zeiss LSM 510 confocal microscope using LSM software.

### Detection of reactive oxygen species

Primary cerebellar cells were plated into 96-well poly-l-lysine–coated black plates (Costar) and cultured for one (neurons) or seven (astrocytes) days prior to overnight incubation with 100 μM d-α-tocopherol (Acros Organics). The following day, the cells were incubated in 10 μg/ml 2',7'-dichlorodihydrofluorescein diacetate in HBSS without phenol red (Invitrogen) for 1 h, washed, and challenged with the indicated concentration of H_2_O_2_ for 1 h. After a final wash in HBSS, DCF fluorescence was measured on a PerkinElmer Victor 3 plate reader (excitation = 485 nm; emission= 535 nm) and normalized to DNA content as measured after incubating cells with bisbenzamide (Sigma; 2.5 μg/ml in 2 M NaCl, 50 mM Na_2_HPO_4_ [pH 7.4] in the dark at 37 °C for 60 min) in a plate reader (excitation = 365 nm; emission = 460 nm).

Statistical analyses and graphing were done using GraphPad Prism 6 (GraphPad Software, Inc). Student's *t* test and one-way ANOVA with Tukey's multiple comparisons post hoc analysis were performed, and threshold for statistical significance was set at *p* < 0.05.

## Data availability

All data are contained within the article.

## Supporting information

This article contains supporting information.

## Conflict of interest

The authors declare that they have no conflicts of interest with the contents of this article.
