# Supplementary material for: The tocopherol transfer protein mediates vitamin E trafficking between cerebellar astrocytes and neurons
Source: J Biol Chem. 2022 Feb 9;298(3):101712. doi: 10.1016/j.jbc.2022.101712 (PMC8913317; doi:10.1016/j.jbc.2022.101712)
Supplement: Supplemental Figure S1 [file mmc1.pdf]

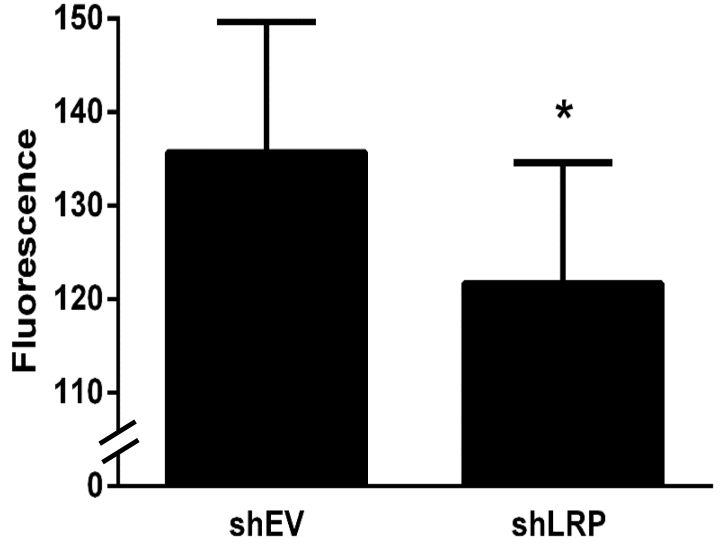

Supplementary Figure 1. LRP participates in vitamin E uptake in differentiated SY5Y cells. SY5Y cells were differentiated to a neuronal phenotype with 10 µM *all-trans* retinoic acid for 7 days and transduced with shEV **(A)** or shLRP1 **(B)** lentiviruses for 72 hours. During the final 24 hours of transduction, cells were loaded with apoE-complexed BODIPY-tocopherol. Fluorescence was visualized in >10 fields by microscopy and normalized to cell protein content. Shown is a representative graph of three independent experiments showing averages and standard deviations of triplicate wells. Asterisks indicate significance of $p < 0.05$ per a Student's t-test.
